# Rapid, Simple and Sensitive Detection of Q Fever by Loop-Mediated Isothermal Amplification of the *htpAB* Gene

**DOI:** 10.1371/journal.pntd.0002231

**Published:** 2013-05-16

**Authors:** Lei Pan, Lijuan Zhang, Desheng Fan, Xiuchun Zhang, Hong Liu, Qunying Lu, Qiyi Xu

**Affiliations:** 1 Dept. of Rickettsiology, National Institute of Communicable Disease Control and Prevention, Chinese Center for Disease Control and Prevention, Beijing, China; 2 YiLi Prefecture Center for Disease Control and Prevention, YiLi, Xinjiang, China; 3 Beijing Center for Disease Control and Prevention, Beijing , China; 4 Centers for Disease Control and Prevention of Anhui Province, Hefei, China; 5 Centers for Disease Control and Prevention of Zhejiang Province, Hangzhou, China; University of California San Diego School of Medicine, United States of America

## Abstract

**Background:**

Q fever is the most widespread zoonosis, and domestic animals are the most common sources of transmission. It is not only difficult to distinguish from other febrile diseases because of the lack of specific clinical manifestations in humans, but it is also difficult to identify the disease in *C. burnetii*-carrying animals because of the lack of identifiable features. Conventional serodiagnosis requires sera from the acute and convalescent stages of infection, which are unavailable at early diagnosis. Nested PCR and real-time PCR require equipment. In this study, we developed a Loop-Mediated Isothermal Amplification **(**LAMP) assay to identify *C. burnetii* rapidly and sensitively.

**Methods:**

A universal LAMP primer set was designed to detect the repeated sequence IS1111a of the *htpAB* gene of *C. burnetii* using PrimerExplorer V4 software. The sensitivity of the LAMP assay was evaluated using known quantities of recombined reference plasmids containing the targeted genes. The specificity of the developed LAMP assay was determined using 26 members of order Rickettsiae and 18 other common pathogens. The utility of the LAMP assay was further compared with real time PCR by the examination 24 blood samples including 6 confirmed and 18 probable Q fever cases, which diagnosed by IFA serological assessment and real time PCR. In addition, 126 animal samples from 4 provinces including 97 goats, 7 cattle, 18 horses, 3 marmots and 1 deer were compared by these two methods.

**Results:**

The limits of detection of the LAMP assay for the *htpAB* gene were 1 copy per reaction. The specificity of the LAMP assay was 100%, and no cross-reaction was observed among the bacteria used in the study. The positive rate of unknown febrile patients was 33.3%(95%CI 30.2%–36.4%) for the LAMP assay and 8.3%(95%CI 7.4%–9.2%) for the real time PCR(P<0.05). Similarly, the total positive rate of animals was 7.9%(95%CI 7.1%–8.7%) for the LAMP assay and 0.8%(95%CI 0.7%–0.9%)for the real time PCR(P<0.01). Using the developed LAMP assay, Q fever in the Yi Li area, Xinjiang Province, was confirmed.

**Conclusions:**

The LAMP assay is a potential tool to support the diagnosis of Q fever in humans and domestic animals in the field, especially in the rural areas of China, because of its rapid and sensitive detection without the aid of sophisticated equipment or a complicated protocol.

## Author Summary

Serological investigations indicate that Q fever is present nationwide in China. However, this zoonosis is poorly understood, and nearly all cases of Q fever are retrospectively diagnosed because of the lack of laboratory diagnosis techniques. Traditional serodiagnosis requires a pair of serum samples from the acute and convalescent stages of infection, which is not useful as an early test of *C. burnetii*. In this study, we developed a rapid, simple and sensitive loop-mediated isothermal amplification (LAMP) assay that targets the *htpAB* gene of *C. burnetii*, in which the lowest limits of the LAMP assay were 1 copy per reaction and the specificity of the LAMP assay was 100%. The sensitivity of the LAMP was significantly higher than that of the real time PCR on evaluation of its utility of detection clinical Q fever patients samples and domestic animals samples. Using the developed LAMP assay, Q fever was first confirmed in the Yi Li area, Xinjiang Province, and the positive rate was 33.3% for unknown febrile patients and 21.0% for domestic animals. Here we concluded that the developed LAMP assay is ideal for the early diagnosis of Q fever, especially in rural areas in China.

## Introduction

Q fever is an important worldwide zoonosis that is caused by *Coxiella burnetii* [1]. The progression of the disease can be either acute or chronic. *C. burnetii* is an obligate intracellular small Gram-negative bacterium that is highly resistant to physical and chemical factors. Q fever can be divided into natural foci and agricultural foci. The former may be transmitted by ticks, while the latter may be transmitted by livestock, including cattle and sheep, and domestic animals, including cats, dogs and rats [2]. Although hard and soft ticks may transmit *C. burnetii* through bites or exposure, Q fever is usually transmitted by aerosol inhalation [3]. Domestic and livestock animals, including cattle, sheep and goats, are regarded as being the most common sources of transmission. When an infected animal gives birth, the amniotic fluid and placenta of the infected animal contaminate the environment, and the pathogen, when present in soil or dust, can survive for a long period of time. The aerosolized bacteria can then be inhaled by humans as a dust particle. In addition, under-processed milk products are also sources of human infection. It has been reported that one bacterium can cause human infection. Thus, *C. burnetii* has been defined as a biological warfare agent by an American anti-terrorist organization. Q fever has become the most widespread zoonosis in the world [4]. *C. burnetii* infection is often misdiagnoses for lacking of typical clinical features. Treatment of Q fever with antibiotics in the acute phase is effective, but once the disease has progressed to its chronic phase, treatment times are longer and relapses are common, resulting in high mortality [5]. In China, Q fever surveillance has been conducted for half a century and has determined that Q fever is widely distributed through China, but all of the studies cases have been retrospective [6], [7]. The greatest challenge to clinicians is diagnosis at the early phase of infection because without proper treatment in the acute phase, acute Q fever will develop into chronic Q fever, and death may result. Conventional serodiagnosis requires sera from both the acute and convalescent stages of infection, which are useless for early diagnosis. Loop-mediated isothermal amplification (LAMP) is a sensitive, specific and simple nucleic acid amplification method that can generate up to 10^9^-fold amplification in less than an hour under isothermal conditions (60–65°C) [8]. The LAMP technique is a potentially rapid and simple diagnostic tool for *C. burnetii* infection. In this study, we developed a rapid, sensitive and specific assay and its utility was compared with the real time PCR previously developed in our laboratory.

## Materials and Methods

### Ethics Statement

The ethics committee of the China CDC reviewed and approved the protocol of the study (No. 201103). All experimental procedures were conducted to conform with institutional guidelines for the care and use of laboratory animals as described by the China CDC, Beijing, China, and confirmed to the National Institutes of Health Guide for Care and Use of Laboratory Animals (Publication No 85-23, revised 1985). A written consent form was obtained before the blood sampling of patients with suspected Q fever. Animal blood sampling was conducted after the animals' owners consented to have the animals involved.

### Bacteria Strains

Of the 42 strains used to determine the specificity of the LAMP assay, 24 samples of the family Rickettsiales and related pathogens were provided by Dr. Raoult D. from the WHO Collaborating Centre for Rickettsial Reference and Research (Marseille, France). *Anaplasma phagocytophilium* strain Webster, MRK, Slovienie and MD were gifted by Dr. JS Dumler at the Johns Hopkins University School of Medicine, and *Ehrlichia chaffeenisis* was provided by Dr. Robert Massung from the U.S. CDC. The use of the above strains was approved by the ethics committee of the China CDC (No.201103), and all samples were anonymized.

### Clinical Samples

Between the years 2007 and 2012, we obtained 24 pairs of blood samples from the acute phase and convalescence of patients with clinical assessment Q fever from Yi Li Prefecture, Xinjiang Province, in 2008. Of these 24 patients, 6 were confirmed to have Q fever by serological analysis (a 4-fold increase in the IgG titer of the antibody against I phase antigen of *C. burnetii* while 18 were laboratory diagnosed of probable Q fever cases (12 patients had single serum IgM positive and IgG positive and 6 patients has single serum IgG positive) [9], the reagents used in the study were from Focus Diagnostics, Cypress, CA 90630 USA and the performance and determination of results were conducted according to the manufacturers' instruction. During the same period, we obtained blood samples from the following animals: horses, marmots, deer, and goats from Yi Li Prefecture, Xinjiang (38 samples, including 18 horses with no clinical manifestations, 3 marmots, 1 deer, and 16 goats, 10 of which were normal and 3 of which were suffering from an unknown illness), Anhui (22 goats, all healthy), Beijing (44 goats, all healthy), and Zhejiang (15 goats and 7 cattle, all healthy). A total of 5 mL of non-anticoagulated blood was collected, and the sera were separated by centrifugation at 444×g for 10 min. The sera were used for serological testing, and the remaining blood clots were used to extract DNA using the QIAamp DNA Mini Kit (Qiagen, Hilden, Germany) according to the manufacturer's instructions. All of the DNA samples from patients and animals were detected by the LAMP assay using 8 µl of the extracted DNA as a template, as described below. To evaluate the utility of the LAMP assay, a real-time PCR assay (SYBR Green I) based on the *htp AB* repetitive element (IS111a) [10], which was previously developed in our lab, was used to test all of the human and animal DNA samples described above. Statistical analysis was conducted using SAS software (version 9.1,SAS Institute, Inc., Cary, NC). Comparison of the positive rates between the two methods was performed using the χ^2^ test and Fisher's exact test. The significance level for these analyses was defined as a P level of 0.05.

### Laboratory Strain DNAs

The specificity of the LAMP assay was determined using a total of 42 strains (Table 1), and their DNA was prepared as follows: the 22 strains of the family Rickettsiales and 2 related pathogens were cultured in L929 cells, the 4 strains of *A. phagocytophilum* were cultured in HL60 cells, and *E. chaffeenisis* was cultured in DH82. Genomic DNA was extracted from cultured cells using the QIAamp DNA Mini Kit (Qiagen, Hilden, Germany). An additional 13 common clinical pathogenic bacteria DNAs were obtained from the relevant departments of the China ICDC (Table 1). In addition, DNA from the blood of healthy humans, cattle, horses, goats and mice was extracted for use as negative controls. Before being used to determine the specificity of the LAMP assay, each bacterial DNA sample was first pre-screened by PCR using universal prokaryotic bacterial 16S rRNA primers [11].

**Table 1:** Laboratory strains for determining the specificity of the LAMP assay.

Classification	Name of pathogen
**Members of the order Rickettsiales**	*Rickettsia prowazekii*, *R. typhi*, *Orientia tsutsugamushi* types karp, kato and Gilliam, *Anaplasma phagocytophilium* type (Webster, MRK, Slovienie and MD), *R. sibirica*, *R. conorii*, *R. honei*, *R. akari*, *R. rickettsii*, *R. africa*, *R. parkeri*, *R. japonica*, *R. slovaca*, *R. aeschlimannii*, *R. montanensis*, *R. helvetica*, *R. felis*, *R. australis*, *R. canadensis*, *R. bellii*, *R. heilongjiangensis*, *Ehrlichia chaffeensis*
**The related species**	*Bartonella henselae ,Bartonella Quintana*
**Other common clinical pathogenic bacteria**	*Coxiella burnetii, Borrelia burgdorferi*, *Escherichia coli*, *Vibrio cholerae*, *Bacillus anthracis*, *Haemophilus influenzae*, *Listeria* spp., *Legionella* spp., *Yersinia pestis*, *Shigella dysenteriae*, *Neisseria meningitides*, *Leptospira* spp., *Borrelia burgdorferi*

### LAMP Primer Design

**Figure 1:**
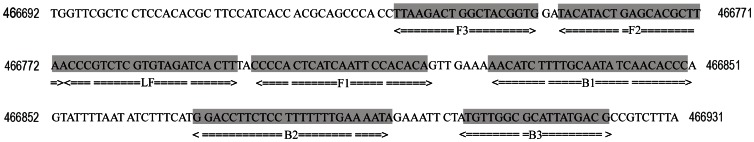
Names and binding sites of primers for Q fever LAMP. The sequences and positions of the set of primers (gray background) against repetitive IS1111a of *htpAB* gene of *C. burnetii* RSA 493 (AE016828.2).

A set of universal primers against the repetitive sequence IS1111a of the *htpAB* gene [12] of *C. burnetii* RSA 493 (AE016828.2) was designed using PrimerExplorer V4 software [http://primerexplorer.jp, Eiken Chemical Co., Ltd., Tokyo, Japan] based on conserved sequences that were determined by aligning 5 *htpAB* GenBank entries (Table 2). Primers were synthesized by Sangon Biotech (Shanghai, China). The primer sequences and their positions relative to the *htpAB* gene are shown in Figure 1.

**Table 2:** Aligned sequences for designing primers and *htpAB* LAMP primer set.

Primer type	Positions	Sequence 5′-3′
**FIP(F1c-F2)**	466797-466818/466754-466773	TGTGTGGAATTGATGAGTGGGG-TACATACTGAGCACGCTTAA
**BIP(B1c-B2)**	466826-466850/466871-466895	AACATCTTTTGCAATATCAACACCC-TATTTTCAAAAAAAGGAGAAGGTCC
**F3**	466734-466751	TTAAGACTGGCTACRGTG
**B3**	466905-466922	CGTCATAATGSGCCAACA
**LF**	466774-466794	AAGTGATCTACACGAGACGGG
**Aligned sequences**	*C. burnetii* RSA 493 (AE016828.2), *C. burnetii* isolate Henzerling RSA 331 *htpAB* transposase gene (DQ882580.1),*C. burnetii* isolate KAV Q154 *htpAB* transposase gene (DQ882594.1), *C. burnetii* isolate M44 *htpAB* transposase gene, partial cds (DQ882595.1), *C. burnetii* isolate WAV *htpAB* transposase gene (DQ882624.1)	

### Reference Plasmid

To determine the sensitivity of the LAMP assay, a recombinant plasmid containing the target sequence of the *C. burnetii* RSA 493 (AE016828.2) *htpAB* gene was constructed. The sequence between the F3 and B3 primer binding sites was amplified using primers QP1 (5′-ACACGCTTCCATCACCACG-3′) and QP2 (5′-TGAAATGGACCCACCCCTT-3′). The resulting PCR products (244 bp) were cloned into the pEASY-T1 vector using the pEASY-T1 Cloning Kit (TransGen, China). The recombinant plasmid was quantified by a NanoPhotometer (Implen, Germany) and was serially diluted (to concentrations of 10^9^, 10^8^, 107, 10^6^, 10^5^, 10^4^, 10^3^, 10^2^, 10^1^, 10^0^ and 10^−1^ copies/µl) to evaluate the limit of detection and the reproducibility of the *htpAB* LAMP assay.

### LAMP Reaction

All LAMP reactions were performed with the Loopamp Kit (Eiken Chemical Co., Ltd., Tokyo, Japan) in a 25 µl reaction volume containing 1.6 µM of each of the FIP and BIP primers, 0.8 µM of the LF and LB primers, 0.2 µM of the F3 and B3 primers, 20 mM Tris–HCl (pH 8.8), 10 mM KCl, 8 mM MgSO_4_, 10 mM (NH4)_2_SO_4_, 0.1% Tween-20, 0.8 M betaine, 1.4 mM of each deoxynucleoside triphosphate (dNTP) and 1 µl *Bst* DNA polymerase (8 U/µl). The reaction was incubated in a real-time turbidimeter (model LA200, Teramecs, Tokyo, Japan) at 63°C for 60 min and then at 80°C for 5 min to terminate the reaction. After amplification, the LAMP products were examined by electrophoresis on 2% agarose gels stained with ethidium bromide or by visual inspection after the addition of 1 µl of 1000X SYBR Green I.

### Evaluation of the Sensitivity and Reproducibility of the LAMP Assay

To compare the sensitivities of the *htpAB* LAMP assay and general PCR, the serially diluted reference plasmid (at concentrations of 10^9^, 10^8^, 107, 10^6^, 10^5^, 10^4^, 10^3^, 10^2^, 10^1^ and 10^0^ copies/µl) containing the target DNA was used to first define the limit of detection. A general PCR with the primers QP1 and QP2 was performed in a 25 µl reaction containing 0.2 µM of each primer, 0.4 mM of each dNTP and 1 U of *Taq* DNA polymerase. The PCR reaction was cycled 35 times using a denaturation step of 94°C for 40 s followed by annealing at 50°C for 30 s and extension at 72°C for 40 s. The PCR products were electrophoresed on a 1% TBE agarose gel and stained with ethidium bromide (1 µg/mL). Genomic DNA from the 44 strains, including the 27 members of the order Rickettsiales, the 3 related agents and the 14 common clinical pathogens, were tested by LAMP to determine the specificity of the *htpAB* LAMP assay. All detection assays were performed in triplicate.

## Results

### Sensitivity and Reproducibility of LAMP

**Figure 2:**
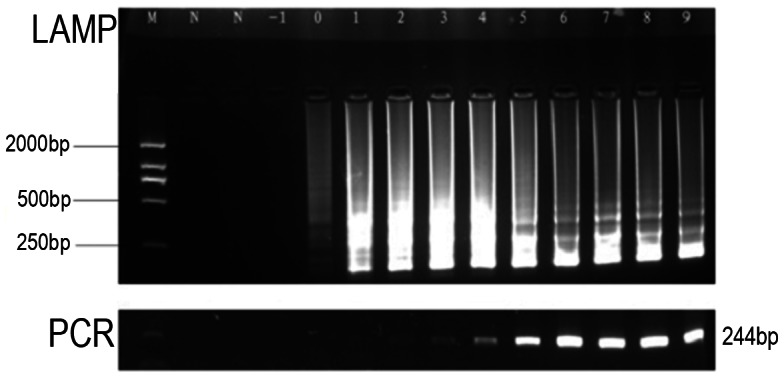
Comparison of the detection limit of LAMP and general PCR. Lane M, DL 2000 bp ladder marker; lanes 10^9^, 10^8^, 10^7^, 10^6^, 10^5^, 10^4^, 10^3^, 10^2^, 10^1^, 10^0^ and 10^−1^ indicate the number of gene copies/µl; lane N, negative control.

The limits of detection of LAMP and PCR for the *htpAB* gene were 1 and 100 copies per reaction, respectively (Figure 2); thus, the LAMP assay is 100-fold more sensitive than conventional PCR at detecting *C. burnetii*. For detection of each concentration of reference plasmid(10^9^, 10^8^, 107, 10^6^, 10^5^, 10^4^, 10^3^, 10^2^, 10^1^ and 10^0^ copies/µl), triplicate reaction results was substantial agreement.

### Specificity of LAMP

To determine the specificity of the LAMP assay for *C. burnetii*, the 27 members of the order Rickettsiales listed in table 1 were tested for amplification. Of these members, a positive result was obtained only for *C. burnetii*. Additionally, the examined common pathogens did not test positive by LAMP. Therefore, these results indicate that the *htpAB* LAMP assay is specific for Q fever.

### Examination of Clinical Samples

When testing 24 blood samples from acute stage of clinical assessment Q fever patients (5 male, age from 29–56 years old; 19 female, age from 16–69 years old) during 2007–2012 , we detected a total of 8 positive results by the LAMP assay and 2 positive by real time PCR. The sensitivity of the LAMP was significant higher than that of the real time PCR(P<0.05)(Table 3). The positive rate of the patients' blood samples was 33.3%(95%CI 30.2%–36.4%) for the LAMP assay and the 8.3%(95%CI 7.4%–9.2%) for the real time PCR detection. Similarly, we detected 126 domestic animals samples by these two assays and the total positive rate of animals was 7.9%(95%CI 7.1%–8.7%) for the LAMP assay and 0.8%(95%CI 0.7%–0.9%)for the real time PCR(P<0.01).

**Table 3:** Results of detection of clinical samples by the LAMP and real-time PCR.

Area	Samples	Positive rate % (No. of positive/No of Negative)	
		LAMP	Real-time PCR
	24 human	33.3(8/24)	8.3(2/24)
	18 horses	22.2(4/18)	0(0/18)
**Xinjiang**	16 goats	25.0(4/16)	6.3(1/16)
	3 marmots	0(0/3)	0(0/3)
	1 deer	0(0/1)	0(0/1)
**Anhui**	22 goats	0 (0/22)	0 (0/22)
**Beijing**	44 goats	4.5(2/44)	0(0/44)
**Zhejiang**	15 goats	0(0/15)	0(0/15)
	7 cattle	0(0/7)	0(0/7)
	Total of animals	7.9(10/126)	0.8(1/126)

Of these 8 positive results by LAMP, 6 confirmed and 18 probable cases of Q fever were diagnosed by the immunofluorescence assay, IFA (6 patients had 4-fold increase in the IgG titer of the antibody against I phase antigen of *C. Burnetii* while 12 patients had single serum IgM and IgG positive and 6 patients has single serum IgG positive). All of the 8 LAMP positive patients reported high fever (≥38°C), anorexia and weakness and had increased hepatic transaminase levels, including AST (61–160 U/L) and ALT (52–201 U/L). Of the 38 animal samples obtained in Yi Li Prefecture, Xinjiang Province, we detected 4 positive goat samples and 4 positive horse samples. No abnormal clinical features were observed in the 4 positive horses. Of the 4 positive goat samples, 3 goats had an illness of unknown origin, and the owner stated that the animals exhibited anorexia, emaciation and lassitude. In addition, 2 goats from Beijing without any clinical manifestations tested positive by LAMP. No positive results were detected in Zhejiang and Anhui Provinces.

## Discussion

Q fever is the most widespread rickettsiosis, and it is difficult to distinguish from other febrile diseases because of a lack of specific clinical manifestations [1]. Due to the lack of recognition and laboratory testing capacity for Q fever, almost all of the recorded cases of Q fever in China are retrospective diagnoses. The most reliable diagnosis for Q fever is the isolation of the pathogen, but such isolation requires cell culture or animal experiments, which are time-consuming, cumbersome and have poor sensitivity. These methods are too dangerous to perform in a normal laboratory and must therefore be performed in a BSL III laboratory. The most common diagnosis method for Q fever is IFA, which tests for a distinctive antibody in the patient's serum. However, IFA is not suitable for early diagnosis because high levels of the appropriate antibody appear only late in the disease progression. Moreover, IFA requires not only specific, expensive instruments that are unavailable in rural areas but it also requires experienced facilities to properly analyze the results. PCR, a simple, sensitive and specific pathogen detection method, has replaced the isolation of pathogens in many cases. Some laboratories have developed nested PCR or semi-nested PCR to detect Q fever, resulting in higher sensitivity and specificity and thereby increasing the detection time and reducing contamination [13], [14]. Real-time PCR has advantages with respect to quantification, control of contamination and sensitivity, but it requires a high-precision thermal cycler [15], [16].

In this study, we developed a sensitive and specific LAMP assay based on the *htpAB* gene [12] of *C. burnetii*. The limit of detection for this assay was 1 copies per reaction. Twenty-seven members of the order Rickettsiales and other pathogenic bacteria were tested, and the results indicate that no cross-reaction occurs among these bacteria. The sensitivity of the LAMP was significant higher than that of the real time PCR on detection 24 human blood samples (33.% vs. 8.3%, P<0.05) and 126 animals samples(7.9% vs. 0.8%, P<0.05).When blood samples from the Yi Li region of Xinjiang Province were analyzed by the LAMP assay, the results indicated that the infection rate was 33.3% for probable cases of human Q fever and 21.0% for domestic animals. Xinjiang is located in the hinterland of the Eurasian continent. It is the province in which the most animal husbandry occurs in China, and livestock breeding is the major agricultural activity. Moreover, more than 50 species of *Ixodid* ticks have been identified in this province [17]. The Yi Li area is located in the northwest of Xinjiang Province. It is an important Central Asian Center, and its foreign boundary is 2,000 km. Its eastern neighbor is Mongolia, its northern neighbor is Russia, and its western neighbor is Kazakhstan. Q fever was previously reported in the southern areas of Xinjiang Province [17]. In 2009, Fan et al. investigated patients with fevers of unknown origin in Yi Li, Xinjiang Province, and found a high IgG titer against *C. burnetii* [9]. The data in the study confirmed that the Yi Li area might be probable foci of Q fever. Unlike scaled-up western modern breeding, the breeding of domestic animals in Xinjiang still involves traditional free grazing. Moreover, Q fever is under-recognized by some local public health organizations such the Centers for Disease Control and Prevention for human or animals, which enhances the risk of the transmission of the *C. burnetii* from animals to human and the risk of an outbreak of zoonotic Q fever in these areas. The differential diagnosis of unknown febrile patients and the empirical determination of specific antibiotics against rickettsiae should be emphasized once-probable cases of Q fever are diagnosed in clinics. In addition, further evaluation of the utility of this LAMP assay and the investigation of the epidemiology of Q fever among domestic animals should select genital tract specimens from animals rather than blood samples to obtain a more accurate rate of *C. burnetii* infection.

In conclusion, we developed a rapid, simple, sensitive and cost-effective LAMP assay to detect *C. burnetii* infection in human and domestic animals. Compared with the real time PCR developed in our laboratory, the LAMP assay is a potential tool to support the diagnosis of Q fever in humans and animals in the field, especially in the rural areas of China because of its rapid and sensitive detection without the aid of sophisticated equipment or complicated operations.

## Supporting Information

Figure S1 **Study design for clinical probable patients with Q fever for the comparative results between the developed LAMP assay and the real time PCR detection.**
(TIF)

Figure S2 **Study design for the domestic animals samples for the comparative results between the developed LAMP assay and the real time PCR detection.**
(TIF)

Figure S3 **The STARD checklist for the reporting of studies of diagnostic accuracy.**
(DOC)
