# Supplementary figures and images for: Rapid, Simple and Sensitive Detection of Q Fever by Loop-Mediated Isothermal Amplification of the htpAB Gene
Source: PLoS Negl Trop Dis. 2013 May 16;7(5):e2231. doi: 10.1371/journal.pntd.0002231 (PMC3656153; doi:10.1371/journal.pntd.0002231)

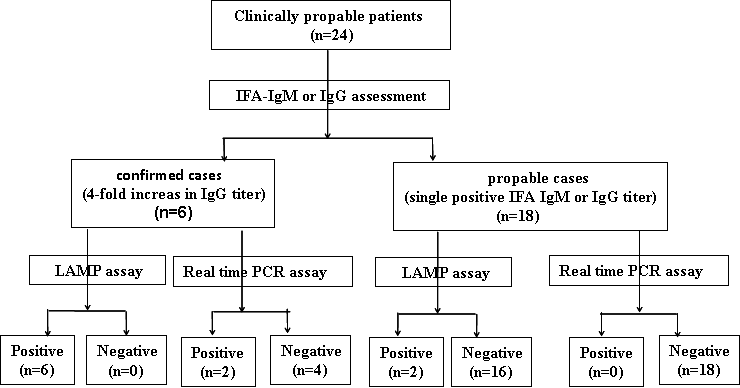

Supplement: Figure S1 — Study design for clinical probable patients with Q fever for the comparative results between the developed LAMP assay and the real time PCR detection. (TIF) [file pntd.0002231.s001.tif]

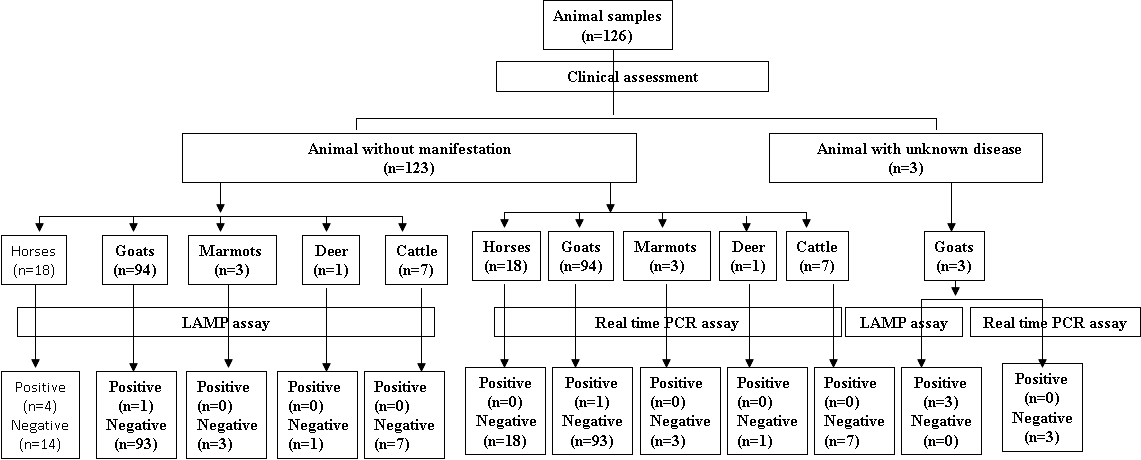

Supplement: Figure S2 — Study design for the domestic animals samples for the comparative results between the developed LAMP assay and the real time PCR detection. (TIF) [file pntd.0002231.s002.tif]
