# Supplementary material for: Rapid, Simple and Sensitive Detection of Q Fever by Loop-Mediated Isothermal Amplification of the htpAB Gene
Source: PLoS Negl Trop Dis. 2013 May 16;7(5):e2231. doi: 10.1371/journal.pntd.0002231 (PMC3656153; doi:10.1371/journal.pntd.0002231)
Supplement: Figure S3 — The STARD checklist for the reporting of studies of diagnostic accuracy. (DOC) [file pntd.0002231.s003.doc]

| **Sectionand Topic** | **Item#** |  | **On page #** |
| --- | --- | --- | --- |
| TITLE/ABSTRACT/ KEYWORDS | 1 | Identify the articleas astudy of diagnostic accuracy (recommend MeSHheading ’sensitivity and specificity’). | 2 |
| INTRODUCTION | 2 | State the research questions or study aims, such as estimating diagnostic accuracy or comparing accuracy betweentests or across participant groups. | 4 |
| METHODS |  |  |  |
| *Participants* | 3 | Describe the study population: The inclusion and exclusion criteria, setting and locations where the data were collected. | 5,6 |
|  | 4 | Describe participant recruitment: Was recruitment based on presenting symptoms, results fromprevious tests, or the fact that the participants hadreceivedthe index tests or the reference standard? | 5,6 |
|  | 5 | Describe participant sampling: Was the study population aconsecutive series of participants defined by the selection criteria in items 3 and 4? If not, specify how participants were further selected. | 5,6 |
|  | 6 | Describe data collection: Was data collection planned before the index test and reference standard were performed (prospective study) or after (retrospective study)? | 5,6 |
| *Test methods* | 7 | Describe the reference standard and its rationale. | 5 |
|  | 8 | Describe technical specifications of material and methods involved including howand when measurements were taken, and/or cite references for index tests and reference standard. | 5 |
|  | 9 | Describe definition of and rationale for the units, cutoffs and/or categories of the results of the index tests and the reference standard. | 5 |
|  | 10 | Describe the number, training and expertise of the persons executing and reading the index tests and the reference standard. | 5,6 |
|  | 11 | Describe whether or not the readers of the index tests andreference standard were blind (masked) to the results of the other test and describe any other clinical information available to the readers. | 5,6 |
| *Statistical methods* | 12 | Describe methods for calculating or comparing measures of diagnostic accuracy, and the statistical methods used to quantify uncertainty (e.g. 95%confidence intervals). |  |
|  | 13 | Describe methods for calculating test reproducibility, if done. |  |
| RESULTS |  |  |  |
| *Participants* | 14 | Report when study was done, including beginning and ending dates of recruitment. | 9 |
|  | 15 | Report clinical and demographic characteristics of the study population (e.g. age, sex, spectrumof presenting symptoms, comorbidity, current treatments, recruitment centers). | 9 |
|  | 16 | Report the number of participants satisfying the criteria for inclusion that did or did not undergo the index tests and/or the reference standard; describe why participants failed to receive either test (a flowdiagramis strongly recommended). | 5,9 |
| *Test results* | 17 | Report time interval fromthe index tests to the reference standard, and any treatment administered between. | 9 |
|  | 18 | Report distribution of severity of disease (define criteria) in those with thetarget condition; other diagnoses inparticipants without the target condition. | 10 |
|  | 19 | Report a cross tabulation of the results of the index tests (including indeterminate and missing results) by the results of the reference standard; for continuous results, the distribution of the test results by the results of the reference standard. | 9 |
|  | 20 | Report any adverse events fromperforming the index tests or the reference standard. | 9,10 |
| *Estimates* | 21 | Report estimates of diagnostic accuracy and measures of statistical uncertainty (e.g. 95%confidence intervals). | 9 |
|  | 22 | Report howindeterminate results, missing responses and outliers of the index tests were handled. |  |
|  | 23 | Report estimates of variability of diagnostic accuracy betweensubgroups of participants, readers or centers, if done. |  |
|  | 24 | Report estimates of test reproducibility, if done. | 9 |
| DISCUSSION | 25 | Discuss the clinical applicability of the study findings. | 11 |

**STARD checklist for the reporting of studies of diagnostic accuracy**
